# Mefenacet [2-(1,3-benzothia­zol-2-yl­oxy)-*N*-methyl-*N*-phenyl­acetamide]

**DOI:** 10.1107/S1600536810044284

**Published:** 2010-11-13

**Authors:** Sanghun Cheon, Tae Ho Kim, Suk-Hee Moon, Jineun Kim

**Affiliations:** a Department of Chemistry and Research Institute of Natural Sciences, Gyeongsang National University, Jinju 660-701, Republic of Korea; b Subdivision of Food Science, Kyungnam College of Information and Technology, Busan 616-701, Republic of Korea

## Abstract

The title compound, C_16_H_14_N_2_O_2_S, crystallizes with two independent mol­ecules in the asymmetric unit. The dihedral angles between the plane of the benzothia­zole ring system and the phenyl ring plane are 51.63 (7) and 60.46 (5)°. In the crystal structure, weak inter­molecular C—H⋯O hydrogen bonds and C—H⋯π inter­actions contribute to the stabilization of the packing.

## Related literature

For information on the toxicity and herbicidal properties of the title compound, see: Lu *et al.* (2001 ▶). For related structures, see: Murru *et al.* (2009 ▶).
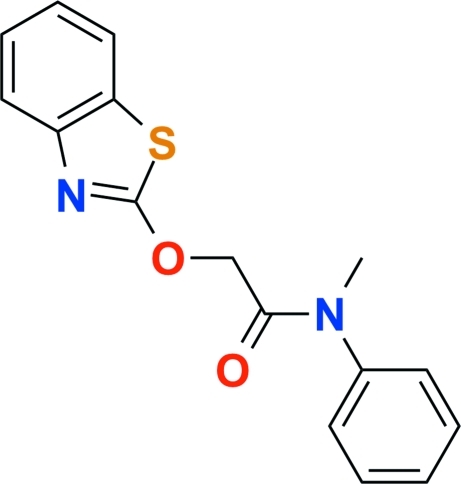


## Experimental

### 

#### Crystal data

C_16_H_14_N_2_O_2_S
*M* *_r_* = 298.35
Monoclinic, 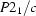

*a* = 11.2708 (6) Å
*b* = 15.7112 (9) Å
*c* = 19.9579 (8) Å
β = 122.997 (2)°
*V* = 2964.0 (3) Å^3^
*Z* = 8
Mo *K*α radiation
μ = 0.22 mm^−1^
*T* = 173 K
0.19 × 0.17 × 0.09 mm

#### Data collection

Bruker APEXII CCD diffractometer
Absorption correction: multi-scan (*SADABS*; Sheldrick, 1996 ▶) *T*_min_ = 0.959, *T*_max_ = 0.980
30345 measured reflections
7338 independent reflections
5174 reflections with *I* > 2σ(*I*)
*R*_int_ = 0.045

#### Refinement

*R*[*F* ^2^ > 2σ(*F* ^2^)] = 0.042
*wR*(*F* ^2^) = 0.107
*S* = 1.02
7338 reflections
381 parameters
H-atom parameters constrained
Δρ_max_ = 0.27 e Å^−3^
Δρ_min_ = −0.28 e Å^−3^

### 

Data collection: *APEX2* (Bruker, 2006 ▶); cell refinement: *SAINT* (Bruker, 2006 ▶); data reduction: *SAINT*; program(s) used to solve structure: *SHELXTL* (Sheldrick, 2008 ▶); program(s) used to refine structure: *SHELXTL*; molecular graphics: *SHELXTL* and *DIAMOND* (Brandenburg, 1998 ▶); software used to prepare material for publication: *SHELXTL*.

## Supplementary Material

Crystal structure: contains datablocks global, I. DOI: 10.1107/S1600536810044284/wn2417sup1.cif

Structure factors: contains datablocks I. DOI: 10.1107/S1600536810044284/wn2417Isup2.hkl

Additional supplementary materials:  crystallographic information; 3D view; checkCIF report

## Figures and Tables

**Table 1:** Hydrogen-bond geometry (Å, °) *Cg*,1 *Cg*2 and *Cg*3 are the centroids of the C27–C32, C17–C22 and S1/C1/C6/N1/C7 rings, respectively.

*D*—H⋯*A*	*D*—H	H⋯*A*	*D*⋯*A*	*D*—H⋯*A*
C2—H2⋯O4^i^	0.95	2.44	3.273 (2)	147
C8—H8*B*⋯O3^ii^	0.99	2.43	3.235 (2)	138
C24—H24*B*⋯O2	0.99	2.35	3.256 (2)	153
C15—H15⋯*Cg*1^ii^	0.95	2.97	3.84	154
C26—H26*B*⋯*Cg*2^iii^	0.98	3.00	3.74	134
C29—H29⋯*Cg*3^iv^	0.95	2.95	3.74	141
C30—H30⋯*Cg*2^i^	0.95	2.80	3.42	124

Symmetry codes: (i) 
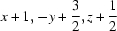
; (ii) 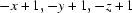; (iii) 
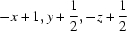
; (iv) 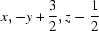.

**Fig. 1.**
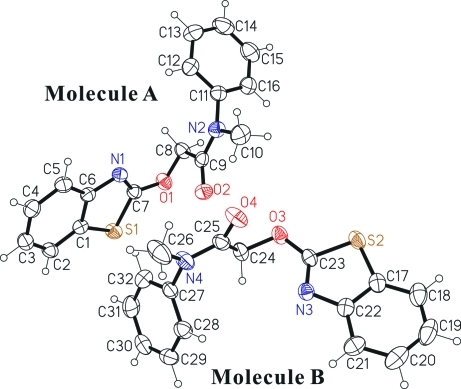
The asymmetric unit of the title compound, with the atom numbering scheme. Displacement ellipsoids are drawn at the 50% probability level. Hydrogen atoms are represented by small spheres of arbitrary radius.

**Fig. 2.**
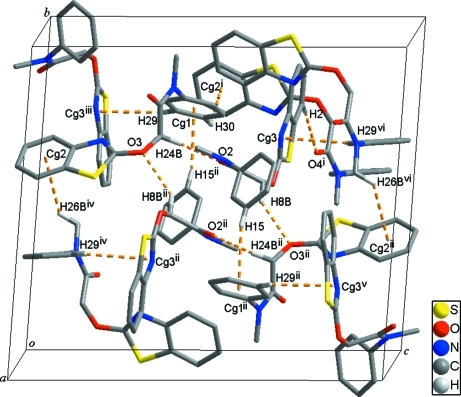
Crystal packing of the title compound with intermolecular C—H···O and C—H···π interactions shown as dashed lines. H atoms not involved in intermolecular interactions have been omitted for clarity. Cg1—Cg3 are the centroids of the C27–C32, C17–C22, and S1/C1/C6/N1/C7 rings, respectively. [Symmetry codes: (i) x + 1, -y + 1.5, z + 1/2; (ii) -x +1, -y + 1, -z + 1; (iii) x, -y + 1.5, z - 1/2; (iv) -x + 1, y - 1/2, -z + 1/2; (v) -x + 1, y - 1/2, -z + 1.5; (vi) x, -y + 1.5, z + 1/2.)

## supplementary crystallographic information

### Comment

Mefenacet (systematic name: 2-(1,3-benzothiazol-2-yloxy)-*N*- methylacetanilide), is a type of herbicide with low toxicity and high activity (Lu *et al.* 2001). However its crystal structure has not hitherto been reported.

In the asymmetric unit (Fig. 1), the dihedral angles between the plane of the benzothiazole ring system and the phenyl ring plane are 51.63 (7)° and 60.46 (5)°. All bond lengths and bond angles are normal and comparable to those observed in similar crystal structures (Murru *et al.,* 2009).

In the crystal structure, as shown in Fig. 2, weak intermolecular C—H···O hydrogen bonds and C—H···π interactions are observed (Table 1). These intermolecular interactions may contribute to the stabilization of the packing.

### Experimental

The title compound was purchased from the Dr. Ehrenstorfer GmbH Company. Slow evaporation of a solution in CH_2_Cl_2_ gave single crystals suitable for X-ray analysis.

### Refinement

All H-atoms were positioned geometrically and refined using a riding model with d(C—H) = 0.95 Å, *U*_iso_(H) = 1.2*U*_eq_(C) for aromatic C, d(C—H) = 0.99 Å, *U*_iso_(H) = 1.2*U*_eq_(C) for CH_2_ and d(C—H) = 0.98 Å, *U*_iso_(H) = 1.5*U*_eq_(C) for CH_3_ groups.

### Crystal data

**Table:**

C_16_H_14_N_2_O_2_S	*F*(000) = 1248
*M**_r_* = 298.35	*D*_x_ = 1.337 Mg m^−^^3^
Monoclinic, *P*2_1_/*c*	Mo *K*α radiation, λ = 0.71073 Å
Hall symbol: -P 2ybc	Cell parameters from 5175 reflections
*a* = 11.2708 (6) Å	θ = 2.2–25.3°
*b* = 15.7112 (9) Å	µ = 0.22 mm^−^^1^
*c* = 19.9579 (8) Å	*T* = 173 K
β = 122.997 (2)°	Block, colourless
*V* = 2964.0 (3) Å^3^	0.19 × 0.17 × 0.09 mm
*Z* = 8	

### Data collection

**Table:**

Bruker APEXII CCD diffractometer	7338 independent reflections
Radiation source: fine-focus sealed tube	5174 reflections with *I* > 2σ(*I*)
graphite	*R*_int_ = 0.045
φ and ω scans	θ_max_ = 28.3°, θ_min_ = 1.8°
Absorption correction: multi-scan (*SADABS*; Sheldrick, 1996)	*h* = −15→15
*T*_min_ = 0.959, *T*_max_ = 0.980	*k* = −18→20
30345 measured reflections	*l* = −26→26

### Refinement

**Table:**

Refinement on *F*^2^	Primary atom site location: structure-invariant direct methods
Least-squares matrix: full	Secondary atom site location: difference Fourier map
*R*[*F*^2^ > 2σ(*F*^2^)] = 0.042	Hydrogen site location: inferred from neighbouring sites
*wR*(*F*^2^) = 0.107	H-atom parameters constrained
*S* = 1.02	*w* = 1/[σ^2^(*F*_o_^2^) + (0.0435*P*)^2^ + 0.7531*P*] where *P* = (*F*_o_^2^ + 2*F*_c_^2^)/3
7338 reflections	(Δ/σ)_max_ = 0.001
381 parameters	Δρ_max_ = 0.27 e Å^−^^3^
0 restraints	Δρ_min_ = −0.28 e Å^−^^3^

### Special details

**Table:**

Geometry. All e.s.d.'s (except the e.s.d. in the dihedral angle between two l.s. planes) are estimated using the full covariance matrix. The cell e.s.d.'s are taken into account individually in the estimation of e.s.d.'s in distances, angles and torsion angles; correlations between e.s.d.'s in cell parameters are only used when they are defined by crystal symmetry. An approximate (isotropic) treatment of cell e.s.d.'s is used for estimating e.s.d.'s involving l.s. planes.
Refinement. Refinement of *F*^2^ against ALL reflections. The weighted *R*-factor *wR* and goodness of fit *S* are based on *F*^2^, conventional *R*-factors *R* are based on *F*, with *F* set to zero for negative *F*^2^. The threshold expression of *F*^2^ > σ(*F*^2^) is used only for calculating *R*-factors(gt) *etc*. and is not relevant to the choice of reflections for refinement. *R*-factors based on *F*^2^ are statistically about twice as large as those based on *F*, and *R*- factors based on ALL data will be even larger.

### Fractional atomic coordinates and isotropic or equivalent isotropic displacement parameters (Å^2^)

**Table:**

	*x*	*y*	*z*	*U*_iso_*/*U*_eq_	
S1	0.94569 (4)	0.65520 (3)	0.70419 (3)	0.03607 (12)	
S2	0.21697 (5)	0.53839 (3)	0.15747 (3)	0.03835 (13)	
O1	0.71887 (12)	0.56299 (8)	0.65599 (8)	0.0371 (3)	
O2	0.53812 (13)	0.61502 (8)	0.50454 (8)	0.0407 (3)	
O3	0.37291 (13)	0.62655 (7)	0.28811 (7)	0.0358 (3)	
O4	0.30217 (13)	0.78901 (8)	0.29306 (9)	0.0462 (3)	
N1	0.69842 (14)	0.70886 (9)	0.66837 (9)	0.0310 (3)	
N2	0.34408 (15)	0.59934 (9)	0.51009 (9)	0.0344 (3)	
N3	0.38648 (14)	0.66403 (9)	0.17928 (8)	0.0310 (3)	
N4	0.52868 (15)	0.83502 (9)	0.36531 (10)	0.0402 (4)	
C1	0.92417 (17)	0.76410 (11)	0.71071 (10)	0.0289 (4)	
C2	1.02172 (17)	0.82906 (12)	0.73152 (10)	0.0338 (4)	
H2	1.1156	0.8173	0.7462	0.041*	
C3	0.97758 (19)	0.91139 (12)	0.73012 (11)	0.0366 (4)	
H3	1.0418	0.9572	0.7434	0.044*	
C4	0.84079 (19)	0.92827 (12)	0.70967 (11)	0.0376 (4)	
H4	0.8133	0.9855	0.7096	0.045*	
C5	0.74394 (18)	0.86369 (11)	0.68950 (11)	0.0356 (4)	
H5	0.6508	0.8760	0.6759	0.043*	
C6	0.78526 (17)	0.78025 (11)	0.68938 (10)	0.0285 (4)	
C7	0.76859 (17)	0.64308 (11)	0.67289 (10)	0.0304 (4)	
C8	0.57044 (18)	0.55577 (11)	0.62402 (11)	0.0349 (4)	
H8A	0.5481	0.5861	0.6593	0.042*	
H8B	0.5454	0.4950	0.6223	0.042*	
C9	0.48359 (18)	0.59301 (10)	0.54072 (10)	0.0311 (4)	
C10	0.2484 (2)	0.62796 (14)	0.42757 (11)	0.0465 (5)	
H10A	0.3006	0.6636	0.4118	0.070*	
H10B	0.1710	0.6610	0.4235	0.070*	
H10C	0.2095	0.5784	0.3922	0.070*	
C11	0.27959 (17)	0.56221 (11)	0.54862 (10)	0.0324 (4)	
C12	0.2600 (2)	0.61034 (13)	0.59977 (12)	0.0417 (4)	
H12	0.2915	0.6677	0.6111	0.050*	
C13	0.1940 (2)	0.57392 (16)	0.63416 (13)	0.0555 (6)	
H13	0.1807	0.6063	0.6698	0.067*	
C14	0.1474 (2)	0.49112 (16)	0.61706 (14)	0.0575 (6)	
H14	0.1009	0.4668	0.6404	0.069*	
C15	0.1676 (2)	0.44331 (14)	0.56647 (14)	0.0540 (6)	
H15	0.1361	0.3859	0.5553	0.065*	
C16	0.2339 (2)	0.47876 (12)	0.53191 (12)	0.0423 (5)	
H16	0.2481	0.4460	0.4968	0.051*	
C17	0.23107 (18)	0.56733 (11)	0.07802 (11)	0.0351 (4)	
C18	0.1640 (2)	0.53245 (13)	0.00209 (13)	0.0489 (5)	
H18	0.1012	0.4856	−0.0126	0.059*	
C19	0.1912 (2)	0.56794 (16)	−0.05143 (14)	0.0589 (6)	
H19	0.1450	0.5458	−0.1041	0.071*	
C20	0.2847 (3)	0.63537 (15)	−0.02975 (13)	0.0555 (6)	
H20	0.3015	0.6584	−0.0679	0.067*	
C21	0.3540 (2)	0.66979 (13)	0.04629 (12)	0.0431 (5)	
H21	0.4188	0.7155	0.0610	0.052*	
C22	0.32599 (18)	0.63536 (11)	0.10061 (11)	0.0323 (4)	
C23	0.33790 (17)	0.61924 (10)	0.21272 (10)	0.0300 (4)	
C24	0.48587 (18)	0.68520 (11)	0.33700 (11)	0.0337 (4)	
H24A	0.5552	0.6842	0.3211	0.040*	
H24B	0.5352	0.6675	0.3937	0.040*	
C25	0.42889 (18)	0.77450 (11)	0.32799 (11)	0.0335 (4)	
C26	0.4871 (2)	0.92358 (13)	0.36470 (17)	0.0638 (7)	
H26A	0.4925	0.9557	0.3244	0.096*	
H26B	0.5510	0.9490	0.4174	0.096*	
H26C	0.3899	0.9252	0.3519	0.096*	
C27	0.67722 (18)	0.81716 (11)	0.40232 (11)	0.0333 (4)	
C28	0.73303 (19)	0.81424 (12)	0.35549 (12)	0.0387 (4)	
H28	0.6737	0.8235	0.2995	0.046*	
C29	0.87550 (19)	0.79774 (12)	0.39043 (12)	0.0416 (5)	
H29	0.9141	0.7954	0.3584	0.050*	
C30	0.9613 (2)	0.78468 (12)	0.47130 (13)	0.0432 (5)	
H30	1.0592	0.7737	0.4953	0.052*	
C31	0.9050 (2)	0.78758 (14)	0.51738 (13)	0.0495 (5)	
H31	0.9646	0.7783	0.5733	0.059*	
C32	0.7627 (2)	0.80379 (13)	0.48354 (12)	0.0455 (5)	
H32	0.7245	0.8057	0.5158	0.055*	

### Atomic displacement parameters (Å^2^)

**Table:**

	*U*^11^	*U*^22^	*U*^33^	*U*^12^	*U*^13^	*U*^23^
S1	0.0286 (2)	0.0329 (2)	0.0472 (3)	0.00576 (18)	0.0210 (2)	0.0021 (2)
S2	0.0339 (2)	0.0301 (2)	0.0484 (3)	−0.00813 (18)	0.0207 (2)	−0.0074 (2)
O1	0.0329 (6)	0.0308 (6)	0.0433 (8)	−0.0001 (5)	0.0178 (6)	0.0011 (6)
O2	0.0446 (7)	0.0475 (8)	0.0369 (7)	−0.0051 (6)	0.0266 (6)	0.0006 (6)
O3	0.0383 (7)	0.0326 (7)	0.0316 (7)	−0.0099 (5)	0.0158 (6)	0.0015 (5)
O4	0.0305 (7)	0.0413 (8)	0.0622 (9)	−0.0038 (6)	0.0224 (7)	0.0042 (7)
N1	0.0274 (7)	0.0329 (8)	0.0328 (8)	0.0009 (6)	0.0166 (6)	−0.0005 (6)
N2	0.0366 (8)	0.0372 (8)	0.0296 (8)	0.0020 (6)	0.0182 (7)	0.0050 (6)
N3	0.0318 (7)	0.0276 (7)	0.0305 (8)	−0.0017 (6)	0.0150 (7)	0.0006 (6)
N4	0.0319 (8)	0.0315 (8)	0.0582 (11)	−0.0043 (6)	0.0251 (8)	−0.0048 (7)
C1	0.0275 (8)	0.0324 (9)	0.0260 (9)	0.0048 (7)	0.0141 (7)	0.0031 (7)
C2	0.0241 (8)	0.0420 (10)	0.0323 (10)	−0.0011 (7)	0.0134 (7)	−0.0003 (8)
C3	0.0364 (9)	0.0349 (10)	0.0366 (10)	−0.0042 (8)	0.0187 (8)	−0.0015 (8)
C4	0.0411 (10)	0.0322 (9)	0.0399 (11)	0.0034 (8)	0.0223 (9)	−0.0004 (8)
C5	0.0293 (9)	0.0365 (10)	0.0417 (11)	0.0053 (7)	0.0199 (8)	−0.0004 (8)
C6	0.0265 (8)	0.0328 (9)	0.0258 (9)	0.0027 (7)	0.0140 (7)	0.0006 (7)
C7	0.0278 (8)	0.0330 (9)	0.0295 (9)	−0.0002 (7)	0.0150 (7)	0.0007 (7)
C8	0.0327 (9)	0.0339 (10)	0.0350 (10)	−0.0049 (7)	0.0165 (8)	0.0027 (8)
C9	0.0384 (9)	0.0250 (8)	0.0311 (9)	−0.0031 (7)	0.0196 (8)	−0.0038 (7)
C10	0.0461 (11)	0.0539 (13)	0.0334 (11)	0.0082 (9)	0.0177 (9)	0.0084 (9)
C11	0.0283 (8)	0.0356 (9)	0.0315 (10)	0.0035 (7)	0.0151 (8)	0.0042 (7)
C12	0.0452 (11)	0.0407 (11)	0.0429 (11)	−0.0015 (9)	0.0264 (10)	−0.0051 (9)
C13	0.0553 (13)	0.0748 (16)	0.0501 (14)	−0.0038 (12)	0.0376 (12)	−0.0081 (12)
C14	0.0472 (12)	0.0788 (17)	0.0521 (14)	−0.0115 (12)	0.0306 (11)	0.0092 (12)
C15	0.0529 (13)	0.0469 (12)	0.0608 (15)	−0.0128 (10)	0.0300 (12)	0.0017 (11)
C16	0.0423 (11)	0.0399 (11)	0.0453 (12)	−0.0020 (8)	0.0241 (10)	−0.0051 (9)
C17	0.0275 (8)	0.0321 (9)	0.0388 (11)	0.0062 (7)	0.0135 (8)	−0.0040 (8)
C18	0.0395 (11)	0.0462 (12)	0.0494 (13)	0.0059 (9)	0.0168 (10)	−0.0157 (10)
C19	0.0596 (14)	0.0667 (15)	0.0420 (13)	0.0114 (12)	0.0223 (11)	−0.0172 (11)
C20	0.0678 (15)	0.0621 (15)	0.0446 (13)	0.0160 (12)	0.0358 (12)	0.0015 (11)
C21	0.0487 (11)	0.0435 (11)	0.0429 (12)	0.0087 (9)	0.0287 (10)	0.0040 (9)
C22	0.0307 (8)	0.0294 (9)	0.0328 (10)	0.0071 (7)	0.0147 (8)	0.0001 (7)
C23	0.0269 (8)	0.0242 (8)	0.0331 (10)	−0.0015 (6)	0.0125 (7)	0.0006 (7)
C24	0.0317 (9)	0.0327 (9)	0.0280 (9)	−0.0070 (7)	0.0107 (8)	0.0016 (7)
C25	0.0325 (9)	0.0352 (10)	0.0351 (10)	−0.0056 (7)	0.0199 (8)	0.0019 (8)
C26	0.0457 (12)	0.0342 (11)	0.114 (2)	−0.0049 (9)	0.0448 (14)	−0.0109 (12)
C27	0.0303 (9)	0.0295 (9)	0.0397 (11)	−0.0070 (7)	0.0187 (8)	−0.0045 (8)
C28	0.0376 (10)	0.0405 (10)	0.0360 (11)	−0.0051 (8)	0.0187 (9)	0.0042 (8)
C29	0.0371 (10)	0.0460 (11)	0.0475 (12)	−0.0053 (8)	0.0268 (10)	0.0003 (9)
C30	0.0320 (10)	0.0363 (10)	0.0516 (13)	−0.0067 (8)	0.0165 (9)	−0.0053 (9)
C31	0.0445 (11)	0.0561 (13)	0.0308 (11)	−0.0048 (10)	0.0095 (9)	−0.0039 (9)
C32	0.0510 (12)	0.0508 (12)	0.0408 (12)	−0.0065 (10)	0.0289 (10)	−0.0090 (9)

### Geometric parameters (Å, °)

**Table:**

S1—C1	1.7428 (17)	C11—C12	1.381 (3)
S1—C7	1.7472 (17)	C12—C13	1.382 (3)
S2—C17	1.738 (2)	C12—H12	0.9500
S2—C23	1.7465 (17)	C13—C14	1.375 (3)
O1—C7	1.344 (2)	C13—H13	0.9500
O1—C8	1.434 (2)	C14—C15	1.374 (3)
O2—C9	1.226 (2)	C14—H14	0.9500
O3—C23	1.336 (2)	C15—C16	1.380 (3)
O3—C24	1.438 (2)	C15—H15	0.9500
O4—C25	1.222 (2)	C16—H16	0.9500
N1—C7	1.274 (2)	C17—C18	1.386 (3)
N1—C6	1.395 (2)	C17—C22	1.402 (2)
N2—C9	1.345 (2)	C18—C19	1.380 (3)
N2—C11	1.438 (2)	C18—H18	0.9500
N2—C10	1.464 (2)	C19—C20	1.387 (3)
N3—C23	1.280 (2)	C19—H19	0.9500
N3—C22	1.403 (2)	C20—C21	1.384 (3)
N4—C25	1.346 (2)	C20—H20	0.9500
N4—C27	1.442 (2)	C21—C22	1.393 (3)
N4—C26	1.466 (2)	C21—H21	0.9500
C1—C2	1.388 (2)	C24—C25	1.512 (2)
C1—C6	1.404 (2)	C24—H24A	0.9900
C2—C3	1.381 (2)	C24—H24B	0.9900
C2—H2	0.9500	C26—H26A	0.9800
C3—C4	1.389 (2)	C26—H26B	0.9800
C3—H3	0.9500	C26—H26C	0.9800
C4—C5	1.380 (3)	C27—C32	1.378 (3)
C4—H4	0.9500	C27—C28	1.383 (2)
C5—C6	1.392 (2)	C28—C29	1.383 (3)
C5—H5	0.9500	C28—H28	0.9500
C8—C9	1.514 (2)	C29—C30	1.372 (3)
C8—H8A	0.9900	C29—H29	0.9500
C8—H8B	0.9900	C30—C31	1.373 (3)
C10—H10A	0.9800	C30—H30	0.9500
C10—H10B	0.9800	C31—C32	1.383 (3)
C10—H10C	0.9800	C31—H31	0.9500
C11—C16	1.381 (3)	C32—H32	0.9500
			
C1—S1—C7	87.44 (8)	C14—C15—C16	119.9 (2)
C17—S2—C23	87.60 (9)	C14—C15—H15	120.0
C7—O1—C8	114.32 (13)	C16—C15—H15	120.0
C23—O3—C24	115.36 (13)	C15—C16—C11	119.62 (19)
C7—N1—C6	108.91 (14)	C15—C16—H16	120.2
C9—N2—C11	122.22 (14)	C11—C16—H16	120.2
C9—N2—C10	119.78 (15)	C18—C17—C22	121.31 (19)
C11—N2—C10	116.67 (14)	C18—C17—S2	128.87 (16)
C23—N3—C22	108.79 (14)	C22—C17—S2	109.82 (13)
C25—N4—C27	122.21 (15)	C19—C18—C17	117.9 (2)
C25—N4—C26	119.92 (16)	C19—C18—H18	121.1
C27—N4—C26	117.75 (15)	C17—C18—H18	121.1
C2—C1—C6	121.92 (16)	C18—C19—C20	121.3 (2)
C2—C1—S1	128.66 (13)	C18—C19—H19	119.4
C6—C1—S1	109.41 (12)	C20—C19—H19	119.4
C3—C2—C1	117.71 (15)	C21—C20—C19	121.3 (2)
C3—C2—H2	121.1	C21—C20—H20	119.3
C1—C2—H2	121.1	C19—C20—H20	119.3
C2—C3—C4	121.00 (17)	C20—C21—C22	118.0 (2)
C2—C3—H3	119.5	C20—C21—H21	121.0
C4—C3—H3	119.5	C22—C21—H21	121.0
C5—C4—C3	121.36 (17)	C21—C22—C17	120.17 (17)
C5—C4—H4	119.3	C21—C22—N3	124.66 (17)
C3—C4—H4	119.3	C17—C22—N3	115.17 (16)
C4—C5—C6	118.74 (16)	N3—C23—O3	126.29 (15)
C4—C5—H5	120.6	N3—C23—S2	118.62 (14)
C6—C5—H5	120.6	O3—C23—S2	115.07 (12)
C5—C6—N1	125.27 (15)	O3—C24—C25	110.61 (14)
C5—C6—C1	119.25 (15)	O3—C24—H24A	109.5
N1—C6—C1	115.47 (14)	C25—C24—H24A	109.5
N1—C7—O1	125.72 (15)	O3—C24—H24B	109.5
N1—C7—S1	118.76 (13)	C25—C24—H24B	109.5
O1—C7—S1	115.52 (12)	H24A—C24—H24B	108.1
O1—C8—C9	110.98 (14)	O4—C25—N4	123.28 (17)
O1—C8—H8A	109.4	O4—C25—C24	121.98 (15)
C9—C8—H8A	109.4	N4—C25—C24	114.64 (15)
O1—C8—H8B	109.4	N4—C26—H26A	109.5
C9—C8—H8B	109.4	N4—C26—H26B	109.5
H8A—C8—H8B	108.0	H26A—C26—H26B	109.5
O2—C9—N2	123.28 (16)	N4—C26—H26C	109.5
O2—C9—C8	121.62 (16)	H26A—C26—H26C	109.5
N2—C9—C8	115.11 (14)	H26B—C26—H26C	109.5
N2—C10—H10A	109.5	C32—C27—C28	120.39 (17)
N2—C10—H10B	109.5	C32—C27—N4	120.35 (16)
H10A—C10—H10B	109.5	C28—C27—N4	119.26 (17)
N2—C10—H10C	109.5	C27—C28—C29	119.74 (18)
H10A—C10—H10C	109.5	C27—C28—H28	120.1
H10B—C10—H10C	109.5	C29—C28—H28	120.1
C16—C11—C12	120.63 (17)	C30—C29—C28	120.13 (18)
C16—C11—N2	119.09 (16)	C30—C29—H29	119.9
C12—C11—N2	120.25 (16)	C28—C29—H29	119.9
C11—C12—C13	119.13 (19)	C29—C30—C31	119.76 (18)
C11—C12—H12	120.4	C29—C30—H30	120.1
C13—C12—H12	120.4	C31—C30—H30	120.1
C14—C13—C12	120.3 (2)	C30—C31—C32	120.97 (19)
C14—C13—H13	119.8	C30—C31—H31	119.5
C12—C13—H13	119.8	C32—C31—H31	119.5
C15—C14—C13	120.40 (19)	C27—C32—C31	119.01 (18)
C15—C14—H14	119.8	C27—C32—H32	120.5
C13—C14—H14	119.8	C31—C32—H32	120.5
			
C7—S1—C1—C2	−178.11 (17)	C23—S2—C17—C18	179.84 (17)
C7—S1—C1—C6	0.69 (13)	C23—S2—C17—C22	0.07 (13)
C6—C1—C2—C3	−0.5 (3)	C22—C17—C18—C19	−1.3 (3)
S1—C1—C2—C3	178.19 (14)	S2—C17—C18—C19	178.94 (15)
C1—C2—C3—C4	0.9 (3)	C17—C18—C19—C20	1.2 (3)
C2—C3—C4—C5	−0.5 (3)	C18—C19—C20—C21	−0.2 (3)
C3—C4—C5—C6	−0.4 (3)	C19—C20—C21—C22	−0.7 (3)
C4—C5—C6—N1	−178.22 (16)	C20—C21—C22—C17	0.6 (3)
C4—C5—C6—C1	0.8 (3)	C20—C21—C22—N3	−179.19 (17)
C7—N1—C6—C5	178.35 (17)	C18—C17—C22—C21	0.4 (3)
C7—N1—C6—C1	−0.7 (2)	S2—C17—C22—C21	−179.77 (13)
C2—C1—C6—C5	−0.4 (3)	C18—C17—C22—N3	−179.76 (15)
S1—C1—C6—C5	−179.29 (13)	S2—C17—C22—N3	0.03 (18)
C2—C1—C6—N1	178.75 (15)	C23—N3—C22—C21	179.63 (16)
S1—C1—C6—N1	−0.15 (19)	C23—N3—C22—C17	−0.2 (2)
C6—N1—C7—O1	−179.10 (16)	C22—N3—C23—O3	178.61 (15)
C6—N1—C7—S1	1.35 (19)	C22—N3—C23—S2	0.22 (18)
C8—O1—C7—N1	5.5 (2)	C24—O3—C23—N3	−6.3 (2)
C8—O1—C7—S1	−174.96 (12)	C24—O3—C23—S2	172.16 (11)
C1—S1—C7—N1	−1.25 (15)	C17—S2—C23—N3	−0.18 (14)
C1—S1—C7—O1	179.15 (14)	C17—S2—C23—O3	−178.75 (13)
C7—O1—C8—C9	69.22 (18)	C23—O3—C24—C25	86.56 (17)
C11—N2—C9—O2	170.94 (16)	C27—N4—C25—O4	−176.47 (17)
C10—N2—C9—O2	4.6 (3)	C26—N4—C25—O4	−0.5 (3)
C11—N2—C9—C8	−8.7 (2)	C27—N4—C25—C24	7.3 (2)
C10—N2—C9—C8	−175.05 (16)	C26—N4—C25—C24	−176.75 (18)
O1—C8—C9—O2	8.8 (2)	O3—C24—C25—O4	10.1 (2)
O1—C8—C9—N2	−171.57 (14)	O3—C24—C25—N4	−173.56 (15)
C9—N2—C11—C16	−87.7 (2)	C25—N4—C27—C32	−98.8 (2)
C10—N2—C11—C16	79.1 (2)	C26—N4—C27—C32	85.1 (2)
C9—N2—C11—C12	94.2 (2)	C25—N4—C27—C28	81.6 (2)
C10—N2—C11—C12	−99.0 (2)	C26—N4—C27—C28	−94.4 (2)
C16—C11—C12—C13	0.0 (3)	C32—C27—C28—C29	0.1 (3)
N2—C11—C12—C13	178.08 (17)	N4—C27—C28—C29	179.57 (16)
C11—C12—C13—C14	−0.6 (3)	C27—C28—C29—C30	−0.3 (3)
C12—C13—C14—C15	0.9 (4)	C28—C29—C30—C31	0.4 (3)
C13—C14—C15—C16	−0.6 (3)	C29—C30—C31—C32	−0.2 (3)
C14—C15—C16—C11	0.1 (3)	C28—C27—C32—C31	0.1 (3)
C12—C11—C16—C15	0.2 (3)	N4—C27—C32—C31	−179.41 (17)
N2—C11—C16—C15	−177.86 (18)	C30—C31—C32—C27	0.0 (3)

### Hydrogen-bond geometry (Å, °)

**Table:**

*Cg*,1 *Cg*2 and *Cg*3 are the centroids of the C27–C32, C17–C22 and S1/C1/C6/N1/C7 rings, respectively.

**Table:**

*D*—H···*A*	*D*—H	H···*A*	*D*···*A*	*D*—H···*A*
C2—H2···O4^i^	0.95	2.44	3.273 (2)	147
C8—H8B···O3^ii^	0.99	2.43	3.235 (2)	138
C24—H24B···O2	0.99	2.35	3.256 (2)	153
C15—H15···Cg1^ii^	0.95	2.97	3.84	154
C26—H26B···Cg2^iii^	0.98	3.00	3.74	134
C29—H29···Cg3^iv^	0.95	2.95	3.74	141
C30—H30···Cg2^i^	0.95	2.80	3.42	124

Symmetry codes: (i) *x*+1, −*y*+3/2, *z*+1/2; (ii) −*x*+1, −*y*+1, −*z*+1; (iii) −*x*+1, *y*+1/2, −*z*+1/2; (iv) *x*, −*y*+3/2, *z*−1/2.
